# THE MEDIATING ROLE OF FATIGUE/SLEEPINESS BETWEEN STATE MINDFULNESS AND SUBJECTIVE COGNITION

**DOI:** 10.1093/geroni/igac059.2416

**Published:** 2022-12-20

**Authors:** Aziz Rehman, Christina Mu, Soomi Lee

**Affiliations:** University of South Florida, Tampa, Florida, United States; University of South Florida, Tampa, Florida, United States; University of South Florida, Tampa, Florida, United States

## Abstract

Previous studies have established a connection between higher mindfulness and cognitive abilities; however, few studies have considered the mechanism underlying this relationship. The cognitive benefit of mindfulness may be through reduced fatigue and daytime sleepiness. This study examined if higher, naturally occurring mindfulness is associated with higher subjective cognition and whether lower fatigue or sleepiness mediate this relationship. Two independent samples of nurses (N1=60 inpatient (IP); N2=84 outpatient (OP)) completed 14 days of ecological momentary assessment (EMA). Fatigue/sleepiness, mindfulness, and subjective cognition (mental speed, processing sharpness, memory) were assessed using EMA. The 5-item Mindful Attention Awareness Scale assessed state mindfulness. Multilevel mediations were conducted in Mplus to account for the nested data. At the within-person level, daily subjective cognition was higher than average on days when mindfulness was higher in both OP and IP samples. This association was mediated by lower levels of fatigue (IP indirect effect: B=2.08, p<.001; OP indirect effect: B=2.57, p<.001) and lower sleepiness (IP indirect effect: B=1.72, p=.001; OP indirect effect: B=0.92, p=.027). The daily indirect pathways were found after controlling for between-person differences; those with higher mindfulness reported higher subjective cognition through lower fatigue, and this effect was only significant in OP nurses (indirect effect: B=11.61, p=.001). Results highlight the importance of monitoring momentary mindfulness and intervening on daily fatigue and sleepiness as these may influence one’s subjective cognition and ultimately their objective performance. These findings may help identify modifiable factors to promote quality of care in nurses and their own well-being.

